# Pulmonary Rehabilitation in Primary Care: Functional and Emotional Impact in a Patient with COPD: A Case Report

**DOI:** 10.3390/reports8040257

**Published:** 2025-12-04

**Authors:** Verónica Esteves, Sara Diogo Gonçalves

**Affiliations:** 1 Unidade de Cuidados na Comunidade de Alijó, Travessa da Tapada, 5070-021 Alijó, Portugal; veroenf@gmail.com; 2 Clinical Academic Center of Trás-os-Montes and Alto Douro (CACTMAD), University of Trás-os-Montes and Alto Douro, 5000-801 Vila Real, Portugal

**Keywords:** chronic obstructive pulmonary disease, pulmonary rehabilitation, primary care, quality of life, case report

## Abstract

**Background and Clinical Significance**: Chronic Obstructive Pulmonary Disease (COPD) is a progressive respiratory disorder characterized by airflow limitation and a significant impact on functional capacity, emotional well-being, and quality of life. Pulmonary rehabilitation improves functional capacity and psychosocial outcomes in individuals with COPD, but evidence on its implementation in primary care is limited. This case report describes the functional and emotional outcomes of a structured pulmonary rehabilitation program delivered in a primary care setting for a patient with moderate COPD, indicating potential feasibility and clinical relevance, while recognizing that evidence from a single case cannot be generalized; **Case Presentation**: A 73-year-old man, an ex-smoker for 10 years, with a history of moderate COPD (GOLD 2/B), controlled hypertension, and recurrent respiratory infections, presented after discontinuation of regular exercise following a previous hospital-based rehabilitation program completed 26 months earlier. The patient reported dyspnea on exertion and functional decline. He completed a 16-week pulmonary rehabilitation program delivered in a primary care setting. The intervention included weekly supervised sessions (breathing exercises, aerobic and resistance training, and education) and twice-weekly home exercises. Outcomes were assessed pre- and post-intervention. Dyspnea improved (mMRC 2 → 1), 6-Minute Walk Test distance increased (303 → 380 m), lower-limb strength improved (10× Sit-to-Stand: 10 → 18 repetitions), perceived exertion decreased (Borg 7 → 4), daily activity limitations were reduced (LCADL 28 → 20), and anxiety decreased (HADS 10 → 6). No adverse events occurred, and adherence was 100%. **Conclusions**: This single case shows that a structured pulmonary rehabilitation program delivered in primary care was feasible and associated with meaningful improvements in functional performance and emotional well-being in a patient with moderate COPD.

## 1. Introduction and Clinical Significance

Chronic Obstructive Pulmonary Disease (COPD) is a progressive respiratory disorder characterized by persistent airflow limitation, chronic symptoms such as dyspnea and cough, and sputum production [1]. Globally, it is the third leading cause of death and affects an estimated 392 million people, with prevalence increasing with age [2]. COPD is associated with reduced exercise tolerance and impaired functional capacity due to ventilatory limitation, skeletal muscle dysfunction, and deconditioning, all of which contribute to substantial declines in daily activity performance and quality of life. Patients frequently experience exacerbations that accelerate disease progression and increase morbidity [3,4].

Pulmonary rehabilitation, a comprehensive and multidisciplinary intervention, is recognized as one of the most effective non-pharmacological strategies in COPD management. It typically combines exercise training, breathing techniques, self-management education, and psychosocial support [5]. Evidence consistently demonstrates its benefits in reducing dyspnea, improving exercise tolerance, enhancing quality of life, and decreasing healthcare utilization [6].

Although pulmonary rehabilitation is well established in specialized or hospital settings, its systematic implementation in primary care remains limited in many regions of Portugal due to organizational constraints, lack of trained personnel, and insufficient infrastructure [7,8]. Recent national reports have emphasized the need to strengthen community-based rehabilitation pathways to improve accessibility and continuity of care.

Clinical Significance: This case report describes the outcomes of a 16-week pulmonary rehabilitation program delivered in a primary care setting for a patient with moderate COPD. While the findings align with existing evidence supporting the benefits of rehabilitation, this report primarily serves as an illustrative example of how such programs can be implemented outside hospital settings. Given the single-case design, conclusions are inherently limited, and no generalization can be inferred.

## 2. Case Presentation

### 2.1. Patient Information

The patient was a 73-year-old man, an ex-smoker with approximately 45 pack-years who quit 10 years earlier. He had a diagnosis of moderate COPD (Global Initiative for Chronic Obstructive Lung Disease (GOLD) 2/B), confirmed by spirometry showing FEV_1_ 53% predicted and DLCO 36% predicted. His comorbidities included controlled hypertension, dyslipidemia, benign prostatic hyperplasia, and recurrent respiratory infections. He reported progressive exertional dyspnea and reduced exercise tolerance following discontinuation of regular physical activity after a hospital-based pulmonary rehabilitation program completed 26 months earlier.

His medication regimen consisted of a long-acting bronchodilator combination (olodaterol + tiotropium), perindopril/amlodipine for hypertension, atorvastatin for dyslipidemia, tamsulosin for benign prostatic hyperplasia, and folic acid supplementation. He lived with his spouse, was independent in activities of daily living, and reported mild anxiety and reduced social participation related to his respiratory symptoms. No relevant family history or genetic conditions were identified.

#### 2.1.1. Medical History

He was diagnosed with moderate COPD (GOLD 2/B), confirmed by spirometry showing an FEV_1_ of 53% predicted and an FEV_1_/FVC ratio consistent with persistent airflow limitation. Diffusing capacity (DLCO) was 36% predicted. His body mass index (BMI) was 22.2 kg/m^2^. Comorbidities included controlled hypertension, dyslipidemia, benign prostatic hyperplasia, and recurrent respiratory infections. He had no history of diabetes or cardiac disease. No additional chronic illnesses relevant to respiratory function were identified. He had no history of diabetes, cardiac disease, or other chronic illnesses and no known allergies.

His chronic medication regimen included a long-acting bronchodilator combination (olodaterol + tiotropium bromide, Spiolto Respimat, 2.5 μg/dose + 2.5 μg/dose), perindopril + amlodipine (10 mg + 5 mg) for hypertension, atorvastatin (20 mg) for dyslipidemia, tamsulosin (0.4 mg) for benign prostatic hyperplasia, and folic acid (5 mg) as a nutritional supplement.

#### 2.1.2. Family History

There was no family history of COPD, asthma, cardiovascular disease, diabetes, or other chronic respiratory, metabolic, or hereditary conditions.

#### 2.1.3. Psychosocial History

The patient was retired, living with his spouse, and independent in activities of daily living. He described social withdrawal related to his breathing limitations and reported mild anxiety symptoms before intervention. He consumed alcohol occasionally in social settings and denied any recreational drug use.

#### 2.1.4. Genetic Information

No relevant genetic testing had been performed, and no hereditary conditions were identified.

#### 2.1.5. Relevant Past Interventions

During the hospital-based pulmonary rehabilitation program, completed 26 months earlier, the patient showed measurable short-term gains, including an improvement in 6-Minute Walk Test distance from 295 m to 340 m and a reduction in dyspnea from Modified Medical Research Council (mMRC) grade 3 to grade 2. However, these gains diminished over time after he discontinued regular exercise, contributing to the functional decline reported at presentation.

### 2.2. Clinical Findings

At baseline, the patient reported exertional dyspnea with an mMRC score of 2. Functional assessments showed a 6-Minute Walk Test distance of 303 m and performance of 10 repetitions on the 10× Sit-to-Stand Test. Perceived exertion during exertion was rated as 7/10 on the Borg scale. Limitations in daily activities were reflected by an London Chest Activity of Daily Living (LCADL) total score of 28. Emotional status was assessed using the Hospital Anxiety and Depression Scale, with an anxiety subscale score of 10. A summary of the patient’s relevant medical history, previous interventions, current presentation, therapeutic plan, and clinical outcomes is provided in Table 1.

### 2.3. Diagnostic Assessment

The diagnosis of COPD was previously established by spirometry according to GOLD criteria. At the time of presentation, the patient was clinically stable with no evidence of exacerbation. No new imaging or laboratory investigations were required beyond functional and symptom assessments. Alternative causes of dyspnea, such as cardiac dysfunction, were not suspected based on clinical history and stable cardiovascular status. Prognostic staging followed GOLD classification (2/B). No diagnostic challenges were encountered.

Cardiac disease was considered a potential contributor to dyspnea; however, the patient had a history of well-controlled hypertension with no known ischemic, valvular, or arrhythmic conditions. He denied chest pain, orthopnea, paroxysmal nocturnal dyspnea, palpitations, or lower-limb edema. Baseline vital signs, cardiovascular examination, and resting oxygen saturation were stable, and no clinical indicators necessitated further cardiac investigation. Given these findings, alternative cardiopulmonary diagnoses were considered unlikely, and no diagnostic challenges were encountered.

### 2.4. Therapeutic Intervention

The intervention consisted of a structured 16-week pulmonary rehabilitation program delivered in a primary care setting. Supervised sessions were held once per week, every Thursday. This frequency was selected to align with community-based feasibility constraints and is consistent with low-intensity, resource-adapted models reported in primary care rehabilitation literature, which combine supervised contact with structured home exercise to maintain training volume and include:

- Breathing exercises: diaphragmatic and pursed-lip breathing performed in seated and standing positions for 10–15 min per session to improve ventilation efficiency and dyspnea control. Supplemental oxygen was not required, and SpO_2_ remained ≥ 90% during training (air to 2 L/min as needed).
- Aerobic training intensity on the treadmill and stationary bicycle was prescribed at 60–70% of the patient’s estimated maximal heart rate (approximately 88–102 bpm) and a target Borg score of 4–6, in accordance with established guidelines for moderate-intensity exercise in COPD rehabilitation. On the treadmill, the selected settings—4 km/h at 12% incline and 5 km/h at 4% incline—corresponded to an estimated workload of 4.5–5.5 METs based on ACSM metabolic equations. Intensity for both treadmill and bicycle training was adjusted weekly based on symptom response (dyspnea and fatigue), oxygen saturation, the patient’s ability to maintain the target Heart rate (HR) range and cadence without distress, and the absence of desaturation.
- Resistance training with elastic bands (Theraband^®^ Gold) followed a structured progression model. Theraband^®^ Gold provides an approximate resistance of 3.9–5.7 kg at 100–200% elongation, based on the manufacturer’s force–elongation data, allowing reproducible load prescription. The patient performed three sets of 15 repetitions for major upper-body muscle groups, maintaining a Borg muscular exertion rating ≤ 5. Progression was introduced when all repetitions were completed with proper technique and without compensatory movements. Progression involved increasing band elongation (thereby increasing resistance within the 3.9–5.7 kg range) or increasing repetitions as tolerated.
- Health education was monitored through a weekly exercise log completed by the patient, documenting frequency, duration, and perceived exertion of each session. Logs were reviewed at every supervised visit, allowing the clinical team to verify adherence, address barriers, and adjust the home program as needed.

Additionally, the patient performed home-based exercises twice per week as instructed. No modifications were made to the protocol, and all sessions were completed as planned. A total of 16 supervised sessions were completed without limitations or adverse events, and the program concluded on 26 August 2025.

The pulmonary rehabilitation program was conducted at a community-based primary care unit within the Local Health Unit of Trás-os-Montes e Alto Douro (northern Portugal). The unit includes a multipurpose exercise room equipped with treadmills, stationary bicycles, elastic resistance bands, and basic monitoring equipment (pulse oximetry, sphygmomanometer, and Borg scale assessment). The sessions were supervised by a physiotherapist with over 10 years of experience in respiratory rehabilitation and a nurse specialized in rehabilitation nursing, both trained in delivering exercise-based interventions for chronic respiratory disease.

On average, the unit provides rehabilitation services to 15–20 patients per month across different conditions (COPD, post-COVID, and musculoskeletal disorders). In contrast to hospital-based programs, this primary care setting emphasizes shorter wait times, continuity of follow-up, and close integration with the patient’s family doctor. However, it operates with limited space and equipment and lacks access to advanced cardiopulmonary monitoring or multidisciplinary teams typical of specialized centers.

### 2.5. Follow-Up and Outcomes

After 16 weeks, the patient demonstrated the following changes in clinical outcomes:

- mMRC dyspnea score improved from 2 to 1.
- 6MWT distance increased from 303 m to 380 m.
- 10× Sit-to-Stand repetitions increased from 10 to 18.
- Borg perceived exertion decreased from 7/10 to 4/10.
- LCADL score improved from 28 to 20.
- HADS anxiety score decreased from 10 to 6.

No adverse events occurred, and adherence to both supervised and home-based sessions was 100%. Adverse events were actively monitored throughout all supervised sessions using continuous symptom reporting, pulse oximetry, and vital-sign checks, and no safety concerns were identified at any point.

The observed improvements were not only numerical but also clinically meaningful. The increase in 6-Minute Walk Test distance (+77 m) exceeded the Minimal Clinically Important Difference (MCID) of 25–35 m for individuals with COPD, indicating a significant gain in functional capacity [9]. Similarly, the reduction in dyspnea (mMRC 2 → 1) surpassed the MCID of 1 point, while the decrease in HADS anxiety score (10 → 6) met the MCID threshold of approximately 1.5–2 points, confirming clinically relevant improvements in both physical and emotional domains. It should be noted that spirometric reassessment was not performed after the intervention, which limits the ability to evaluate physiological changes alongside functional improvements. In addition, the follow-up period was short, limiting the ability to determine whether the observed improvements would be sustained over time, particularly in chronic conditions such as COPD, where functional gains often diminish without continued support.

## 3. Discussion

### 3.1. Scientific Rationale

The observed improvements are consistent with physiological responses typically associated with aerobic and resistance training in individuals with COPD; however, given the single-case design, no causal inferences can be drawn. The combination of supervised exercise, breathing strategies, and education may have contributed to the patient’s increased confidence and activity tolerance, but these associations should be interpreted cautiously. The changes observed in this case align with patterns reported in previous studies, though controlled research is required to determine the extent to which specific components of the intervention influence functional or emotional outcomes. Although the 77 m increase in 6MWT distance exceeds the established MCID for COPD, potential confounders must be considered. A modest learning effect between baseline and follow-up tests, and increased patient motivation during the post-intervention assessment, may have contributed to the observed improvement [10]. However, the magnitude of change exceeds that typically attributed to learning effects alone, suggesting a true functional gain.

Although the patient demonstrated clear improvements across functional and emotional outcomes, these changes cannot be attributed solely to the rehabilitation program. As this is a single-case report, natural day-to-day variability in performance, fluctuations in symptom perception, increased familiarity with testing procedures, and regression to the mean may also have contributed to the observed changes. Therefore, the findings should be interpreted as descriptive rather than causal.

### 3.2. Comparison with the Literature

The observed improvements are consistent with previous evidence demonstrating that pulmonary rehabilitation reduces dyspnea, increases exercise tolerance, and improves quality of life in patients with COPD [11,12,13]. International studies and recent Portuguese reports highlight clinically significant increases in 6MWT distance and reductions in symptom burden following rehabilitation programs [14]. The decrease in anxiety symptoms aligns with the established psychological benefits of structured exercise and education in chronic respiratory disease [15,16]. What distinguishes this case is the observation of similar improvements in a primary care setting, suggesting a possible role for community-based rehabilitation delivery. However, broader studies are required to confirm this.

In contrast to hospital-based pulmonary rehabilitation, which typically benefits from larger multidisciplinary teams, advanced cardiopulmonary monitoring equipment, and structured group programs, primary-care rehabilitation operates within smaller teams and more limited infrastructure [17,18]. Primary-care settings often offer greater accessibility, shorter waiting times, and closer coordination with family physicians, facilitating continuity of follow-up and integration into routine chronic disease management [19]. However, these settings may lack specialized facilities, have fewer dedicated rehabilitation staff, and rely on simpler monitoring tools, requiring programs to be adapted to the available resources [20,21]. In this case, the primary-care environment allowed for personalized supervision by a physiotherapist and a rehabilitation nurse, with a focus on individualized exercise progression rather than the more standardized protocols often used in hospital programs. These contextual differences may influence patient adherence, feasibility, and the scope of rehabilitation delivered. This case provides a preliminary example of the possibility of such integration. It suggests that primary care could play a more active role in long-term COPD management and rehabilitation follow-up, although broader evidence is needed before drawing firm conclusions.

Although conclusions from a single case cannot be generalized, this report offers a preliminary observation that aligns with emerging evidence suggesting that pulmonary rehabilitation may be feasible to implement in primary care settings. Future prospective studies with larger cohorts are essential to confirm these preliminary observations and to evaluate long-term outcomes.

### 3.3. Strengths and Limitations

This case demonstrates the feasibility and benefits of delivering a structured pulmonary rehabilitation program in a primary care setting. Strengths include the comprehensive assessment of functional, emotional, and daily activity outcomes using validated tools, as well as the integration of supervised and home-based training to encourage long-term adherence. Another strength is the patient-centered approach, which emphasizes accessibility and continuity of care close to the patient’s home, potentially increasing motivation compared with previous hospital-based rehabilitation [22].

Limitations include the single-patient design, which precludes generalization of results. The relatively short follow-up period does not allow for conclusions about the long-term maintenance of the observed benefits. Furthermore, the absence of post-intervention spirometry limits the assessment of physiological improvement and its relationship to functional outcomes. Future studies should incorporate objective pulmonary measures and extended follow-up to better understand the durability and physiological correlates of these changes.

### 3.4. Patient Perspective

The patient expressed satisfaction with the program, noting that supervised sessions increased his confidence in exercising safely and helped him maintain regular practice. He reported meaningful improvements in breathing, physical ability, and emotional well-being. He also indicated that receiving rehabilitation within primary care, in a familiar and easily accessible environment, supported consistent participation compared with his earlier hospital-based program.

### 3.5. Primary Takeaway Lessons

This case illustrates that pulmonary rehabilitation delivered in primary care can achieve significant functional and emotional benefits for patients with COPD. Individualized, structured, and accessible programs that combine supervised and home-based training may enhance adherence and extend the reach of rehabilitation beyond specialized hospital settings. This model of care offers a promising avenue for improving the quality of life and independence in individuals living with chronic respiratory disease.

## 4. Conclusions

This case report highlights the observed improvements associated with a structured pulmonary rehabilitation program delivered in a primary care setting. The findings suggest potential benefits for dyspnea, functional capacity, daily activities, and emotional well-being. While encouraging, these observations are based on a single case and cannot be interpreted as evidence of effectiveness. Nonetheless, the case illustrates how primary-care–based rehabilitation may provide a feasible and accessible approach to supporting individuals living with COPD.

## Figures and Tables

**Table 1:** Timeline of patient history, assessments, intervention, and outcomes.

Date/Interval	Category	Description
26 months prior	Clinical Event	Completed hospital-based pulmonary rehabilitation program; discontinued exercise afterward.
Baseline (Week 0)	Clinical Event	Presentation at the primary care unit with exertional dyspnea and functional decline.
Baseline (Week 0)	Assessments	mMRC: 2; 6MWT: 303 m; 10× Sit-to-Stand: 10 reps; Borg (exertion): 7/10; LCADL: 28; HADS-Anxiety: 10.
Weeks 1–16	Intervention	Weekly supervised pulmonary rehabilitation sessions (breathing exercises, aerobic training, resistance training, education) + twice-weekly home-based exercises
Week 16	Post-intervention Assessments	mMRC: 1; 6MWT: 380 m; 10× Sit-to-Stand: 18 reps; Borg: 4/10; LCADL: 20; HADS-Anxiety: 6.
Week 16	Outcomes	Improved functional capacity, reduced dyspnea, fewer daily limitations, reduced anxiety; 100% adherence; no adverse events.

6MWT: 6-Minute Walk Test; HADPS: Hospital Anxiety and Depression Scale; mMRC: Modified Medical Research Council.

## Data Availability

Data supporting the findings and conclusions are available on request from the corresponding author.

## Abbreviations

The following abbreviations are used in this manuscript:

6-MWT	6-Minute Walk Test
COPD	Chronic Obstructive Pulmonary Disease
HADS	Hospital Anxiety and Depression Scale
LCADL	London Chest Activity of Daily Living Scale
mMRC	Modified Medical Research Council

## References

[1] Jo Y.S. (2022). Long-Term Outcome of Chronic Obstructive Pulmonary Disease: A Review. Tuberc. Respir. Dis..

[2] Adeloye D., Song P., Zhu Y., Campbell H., Sheikh A., Rudan I. (2022). Global, Regional, and National Prevalence of, and Risk Factors for, Chronic Obstructive Pulmonary Disease (COPD) in 2019: A Systematic Review and Modelling Analysis. Lancet Respir. Med..

[3] Ursoleo J.D., Bottussi A., Sullivan D.R., D’Andria C., Smirnova N., Rosa W.E., Nava S., Monaco F. (2025). Chronic Obstructive Pulmonary Disease: A Narrative Synthesis of Its Hallmarks for Palliative Care Clinicians. Eur. J. Intern. Med..

[4] Von Leupoldt A., Fritzsche A., Trueba A.F., Meuret A.E., Ritz T. (2012). Behavioral Medicine Approaches to Chronic Obstructive Pulmonary Disease. Ann. Behav. Med..

[5] Arnold M.T., Dolezal B.A., Cooper C.B. (2020). Pulmonary Rehabilitation for Chronic Obstructive Pulmonary Disease: Highly Effective but Often Overlooked. Tuberc. Respir. Dis..

[6] Morris J.R., Harrison S.L., Robinson J., Martin D., Avery L. (2023). Non-Pharmacological and Non-Invasive Interventions for Chronic Pain in People with Chronic Obstructive Pulmonary Disease: A Systematic Review without Meta-Analysis. Respir. Med..

[7] Ferreira A., Vigia C., Ferreira R., Severino S., Guerra N., Helena J., Sousa L. (2025). Respiratory Rehabilitation Programs in Portugal: Rapid Review. Rehabil. Interdiscip..

[8] Silva L., Mota Â., Lemos L., Santos M., Cunha H., Maricoto T., Costa P., Padilha M. (2024). Characterisation of Patients With Chronic Obstructive Pulmonary Disease (COPD) From an Urban Municipality in the Northern Region of Portugal: A Cross-Sectional Study. Cureus.

[9] Wise R.A., Brown C.D. (2005). Minimal Clinically Important Differences in the Six-Minute Walk Test and the Incremental Shuttle Walking Test. COPD J. Chronic Obstr. Pulm. Dis..

[10] Pedersen C., Halvari H., Williams G.C. (2018). Worksite Intervention Effects on Motivation, Physical Activity, and Health: A Cluster Randomized Controlled Trial. Psychol. Sport Exerc..

[11] McCarthy B., Casey D., Devane D., Murphy K., Murphy E., Lacasse Y. (2015). Pulmonary Rehabilitation for Chronic Obstructive Pulmonary Disease. Cochrane Database Syst. Rev..

[12] Daabis R., Hassan M., Zidan M. (2017). Endurance and Strength Training in Pulmonary Rehabilitation for COPD Patients. Egypt. J. Chest Dis. Tuberc..

[13] Vendrell L.V., Islas D.G., Tejeda A.O., Ramírez M.E.Q., Santillan R.N.S., Amor S.G., Hernández J.C.G., Mellado E.T., Sánchez I.A.S., Reyna O.U.M. (2020). Effect of Pulmonary Rehabilitation on Exercise Tolerance and Functional Class in Patients with COPD and Heart Failure. Eur. Respir. J..

[14] Agarwala P., Salzman S.H. (2020). Six-Minute Walk Test. Chest.

[15] Farver-Vestergaard I., Buksted E.H., Sørensen D., Jonstrup S., Hansen H., Christiansen C.F., Løkke A. (2024). Changes in COPD-Related Anxiety Symptoms during Pulmonary Rehabilitation: A Prospective Quantitative and Qualitative Study. Front. Rehabil. Sci..

[16] Almdabgy E.M., Qader A., Binjahlan A.A., Alshalawi A.M., Albeladi A., Alharbi W.S., Almehmadi K.A. (2023). The Impact of Pulmonary Rehabilitation on Mental Health and Quality of Life in Patients With Chronic Obstructive Pulmonary Disease (COPD): A Narrative Review. Cureus.

[17] Qin H., Jia P., Yan Q., Li X., Zhang Y., Jiang H., Yang H., Li L. (2025). Barriers and Facilitators to Pulmonary Rehabilitation in COPD: A Mixed-Methods Systematic Review. BMC Pulm. Med..

[18] Cui S., Zhang S., Ren H., Zhang Y., Geng L. (2025). Efficacy of Multidisciplinary Team-Based Cardiopulmonary Rehabilitation in Improving Outcomes for Patients with Intensive Care Unit-Acquired Weakness: A Retrospective Cohort Study. J. Multidiscip. Healthc..

[19] Cox N.S., Oliveira C.C., Lahham A., Holland A.E. (2017). Pulmonary Rehabilitation Referral and Participation Are Commonly Influenced by Environment, Knowledge, and Beliefs about Consequences: A Systematic Review Using the Theoretical Domains Framework. J. Physiother..

[20] Chen P., Zanca J., Esposito E., Barrett A.M. (2021). Barriers and Facilitators to Rehabilitation Care of Individuals With Spatial Neglect: A Qualitative Study of Professional Views. Arch. Rehabil. Res. Clin. Transl..

[21] Bickenbach J., Stucki G. (2021). Supporting Evidence-Informed Policy Making in Rehabilitation: A Logic Framework for Continuous Improvement of Rehabilitation Programs. Health Policy.

[22] Edgman-Levitan S., Schoenbaum S.C. (2021). Patient-Centered Care: Achieving Higher Quality by Designing Care through the Patient’s Eyes. Isr. J. Health Policy Res..

